# Pulmonary Embolism and Increased Levels of D-Dimer in Patients with Coronavirus Disease

**DOI:** 10.3201/eid2610.202127

**Published:** 2020-10

**Authors:** Kok Hoe Chan, Jihad Slim, Hamid S. Shaaban

**Affiliations:** St. Michael’s Medical Center, Newark, New Jersey, USA

**Keywords:** COVID-19, cytokine storm, Factor XIII deficiency, interleukin-6 receptor, plasminogen activator, proteolytic enzymes, tocilizumab, transient hypercoagulable state, venous thromboembolism, coronavirus disease, respiratory infections, viruses, severe acute respiratory syndrome coronavirus 2, SARS-CoV-2

**To the Editor:** We read with great interest the recent report by Griffin et. al. (*1*). Griffin et al. reported on 3 patients in whom pulmonary embolism developed after the cytokine storm phase of coronavirus disease (COVID-19); the patients were treated with steroids and tocilizumab. We have observed a transient elevation of d-dimer in patients after tocilizumab treatment, which leads to an interesting discussion about whether the pulmonary embolism observed in these COVID-19 patients was due to a persistent hypercoagulable state in the late phase of the disease or a transient one related to tocilizumab. 

Tocilizumab is a humanized antihuman interleukin-6 (IL-6) receptor monoclonal antibody that inhibits IL-6 signaling. Use of tocilizumab in the COVID-19 pandemic has been growing. It presumptively targets the cytokine storm phase of the disease by inhibiting the IL-6 pathway (*2*). However, IL-6 has a multifaceted role in venous thromboembolism, and Zhang et al. has reported that upregulation of IL-6 as the result of aberrant downregulation of miR-338-5p may lead to venous thromboembolism (*3*). 

Conversely, using a rat model, Nosaka et al. demonstrated the importance of iIL-6 in resolving thrombi through macrophage recruitment and proteolytic enzymes induction (*4*). The absence of IL-6, in fact, leads to the thrombus growing (*4*). Moreover, tocilizumab has been reported to decrease factor XIII, chemerin, and plasminogen activator inhibitor levels (*5*). Factor XIII is involved in fibrin stabilization; blocking this factor may lead to fibrin clot instability, causing microthrombi to dislodge, increasing the likelihood of thrombophilia. 

The association of tocilizumab with thrombosis is not clearly understood. However, the potential for adverse effects that we describe may warrant a short period of therapeutic anticoagulation before and after administering tocilizumab. The hypercoagulable state reported in the findings by Griffin et. al. may represent a side effect of tocilizumab rather than being a condition secondary to COVID-19, or it could result from a combination of both.
